# Modeling and mining term association for improving biomedical information retrieval performance

**DOI:** 10.1186/1471-2105-13-S9-S2

**Published:** 2012-06-11

**Authors:** Qinmin Hu, Jimmy Xiangji Huang, Xiaohua Hu

**Affiliations:** 1 Information Retrieval and Knowledge Management Research Lab, York University, Toronto, ON, M3J1P3, Canada; 2 Department of Computer Science & Engineering, York University, Toronto, ON, M3J1P3, Canada; 3 School of Information Technology, York University, Toronto, ON, M3J1P3, Canada; 4 College of Information Science and Technology, Drexel University, Philadelphia, PA, 19104, USA

## Abstract

**Background:**

The growth of the biomedical information requires most information retrieval systems to provide short and specific answers in response to complex user queries. Semantic information in the form of free text that is structured in a way makes it straightforward for humans to read but more difficult for computers to interpret automatically and search efficiently. One of the reasons is that most traditional information retrieval models assume terms are conditionally independent given a document/passage. Therefore, we are motivated to consider term associations within different contexts to help the models understand semantic information and use it for improving biomedical information retrieval performance.

**Results:**

We propose a term association approach to discover term associations among the keywords from a query. The experiments are conducted on the TREC 2004-2007 Genomics data sets and the TREC 2004 HARD data set. The proposed approach is promising and achieves superiority over the baselines and the GSP results. The parameter settings and different indices are investigated that the sentence-based index produces the best results in terms of the document-level, the word-based index for the best results in terms of the passage-level and the paragraph-based index for the best results in terms of the passage2-level. Furthermore, the best term association results always come from the best baseline. The tuning number *k *in the proposed recursive re-ranking algorithm is discussed and locally optimized to be 10.

**Conclusions:**

First, modelling term association for improving biomedical information retrieval using factor analysis, is one of the major contributions in our work. Second, the experiments confirm that term association considering co-occurrence and dependency among the keywords can produce better results than the baselines treating the keywords independently. Third, the baselines are re-ranked according to the importance and reliance of latent factors behind term associations. These latent factors are decided by the proposed model and their term appearances in the first round retrieved passages.

## Background

The use of large-scale experimental techniques and biomedical tools has increased the pace at which biologists produce useful information. This also promotes the growth of the scientific literature, which contains information on those experimental results in the form of free text that is structured in a way which makes it straightforward for humans to read but more difficult for computers to interpret automatically and search efficiently. As a consequence, there is increasing interest in methods that can handle collections of biomedical texts. Such methods include systems that efficiently retrieve and classify information in response to complex user queries, and beyond this, systems that carry out a deeper analysis of the literature to extract specific associations.

Information retrieval (IR) deals with text analysis, text storage, and the retrieval of stored records having similarity between them [1]. In context of biomedical domain, IR systems are to retrieve documents/passages that a user might find relevant to his or her information need. What many information seekers, really desire to be provided short, specific answers to questions and put them in context by providing supporting information and linking to original sources [2]. There are situations when the terms retrieved by IR systems, are not the only desirably independent but associations among the terms within different contexts or a single text, which provide an insight into the text as answers, might be of interest in some specific domains like biomedical domain, text summarization, question answering systems and so on. 

In this paper, we focus on discovering term associations among the keywords from a query. Taking all the keywords as a sequence, we consider some subsequences as terms and propose a factor analysis based model to provide knowledge for finding the importance of term associations statistically. In our scientific fields, variables such as "intelligence" or "leadership quality" can not be measured directly. Such variables, called latent variables, can be measured by other "quantifiable" variables, which reflect the underlying variables of interest. Factor analysis attempts to explain the correlations between the observed term associations in terms of the underlying factors, which are not directly observable. These latent factors can be considered the same as the hidden variables of "eliteness" introduced by Robertson et al [3] in order to gain some understanding of the relation among multiple term occurrences and relevance. The observations for the proposed approach can be obtained from the keywords that are extracted from the queries, and from the passages retrieved by an IR system. In order to find the latent factors for term associations, we compute the factor loadings [4] using MATLAB [5]. Then we calculate the communalities [4] based on factor loadings to indicate the importance and reliance of latent factors and use them to recursively re-rank the baseline result for improving retrieval performance. In addition, in order to evaluate the superiority of the proposed approach, the generalized sequential pattern (GSP) algorithm is adopted as a comparison. 

The paper is organized as follows. First, we briefly present the experimental results and discussions in the results and discussion section, where the IR environment is introduced with the descriptions of the data sets, queries, evaluation measures, the IR system and indices. The comprehensive empirical study includes the analysis for the baselines, the proposed term association, the influence of different indices and **k **for the recursive re-ranking algorithm, the comparisons to the GSP algorithm and the official submissions. Second, we show our contributions in the conclusion section. Third, in the methods section, we propose our methods systematically and consistently. A term association approach is presented, followed by a factor analysis based model and a corresponding algorithm, including a recursive re-ranking algorithm. The related work is also presented in this section.

## Results and discussions

Here we report the results obtained from a set of experiments conducted on the TREC 2004-2007 Genomics data sets and 2004 HARD data set, in order to evaluate the effectiveness of the proposed model and algorithms.

### Experimental environment

#### Data sets and queries

We evaluate the proposed model and algorithms on the TREC 2004-2007 Genomics data sets, since we focus on the biomedical domain. Furthermore, we also apply the TREC 2004 HARD data set for evaluation.

**TREC 2007 and 2006 Genomics data sets **provide a test collection of 162,259 full-text documents assembled with 36 queries in 2007 and 28 queries in 2006. The TREC 2007 queries are in the form of questions asking for lists of specific entities. The definitions for these entity types are based on controlled terminologies from different sources, with the source of the terms depending on the entity type [6]. The TREC 2006 queries are derived from the set of biologically relevant questions based on the Generic Topic Types (GTTs) [2]. All these queries are listed on the official genomics website at: http://ir.ohsu.edu/genomics.

**TREC 2005 and 2004 Genomics data sets **consists of a document collection for the ad hoc retrieval task which is a 10-year subset of MEDLINE with completed citations from the database inclusive from 1994 to 2003. This provides a total of 4,591,008 records [2]. Each record is an abstract of a document. Then in this paper, we take an abstract as a passage. There are 50 queries for each year respectively. More information can be found at: http://www.ncbi.nlm.nih.gov/.

**TREC 2004 HARD data set **consists of entirely of English text, such as the Agence France Press (AFP), Associated Press (APW), Central News Agency (CNA), LA Times/Wash Post (LAT), New York Times (NYT), Salon.com (SLN), Ummah Press (UMM), Xinhua English (XIN) with the total collection of 652,710 documents. In our research, we parse the documents into passages [7]. There are 25 queries used in this paper.

#### Evaluation measures

The TREC Genomics Track has three evaluation measures that are the document-level, the aspect-level and the passage2-level (a new measure for the TREC 2007 queries) [6]. Each of these provides insight into the overall performance for a user trying to answer the given queries and measured by some variant of mean average precision (MAP), which are briefly described as follows.

##### Document-level

This is a standard IR measure. The precision is measured at every point where a relevant document is obtained and then averaged over all relevant documents to obtain the average precision for a given query. For a set of queries, the mean of the average precision for all queries is the mean average passage precision of that IR system.

##### Passage-level

As described in [8], this is a character-based precision calculated as follows. For each relevant retrieved passage, precision will be computed as the fraction of characters overlapping with the gold standard passages divided by the total number of characters included in all nominated passages from this system for the topic up until that point. Similar to regular MAP, relevant passages that are not retrieved will be added into the calculation as well, with precision set to 0 for relevant passages not retrieved. Then the mean of these average precisions over all topics will be calculated to compute the mean average passage precision.

##### Passage2-level

This is a new character-based MAP measure which is added to compare the accuracy of the extracted answers and modified from the original measure Passage MAP. Passage2 treats each individually retrieved character in published order as relevant or not, in a sort of "every character is a mini relevance-judged document" approach [6]. This is done to increase the stability of the passage MAP measure against arbitrary passage splitting techniques.

#### Gold standard

A gold standard is created by extracting out the relevance passages and entities for each topic. Judges for the relevant passages and entities are recruited from the institutions of track participants and other academic or research centres. They are required to have significant domain knowledge, typically in the form of a PhD in a life science. In summary, judges are given the following three instructions. First, reviewing the topic question and identifying key concepts. Second, identifying relevant paragraphs and selecting minimum complete and correct excerpts. Third, developing controlled vocabulary for entities based on the relevant passages and coding entities for each relevant passage based on this vocabulary [8].

#### System

We used Okapi BSS (Basic Search System) as our main search system. Okapi is an information retrieval system based on the probability model of Robertson and Sparck Jones [3,9-14]. The retrieval documents are ranked in the order of their probabilities of relevance to the query. Search term is assigned weight based on its within-document term frequency and query term frequency. The weighting function used is BM25.

$$\begin{array}{c} w=\frac{\left(k_{1}+1\right)*tf}{K+tf}*\text{log}\frac{\left(r+0.5\right)/\left(R-r+0.5\right)}{\left(n-r+0.5\right)/\left(N-n-R+r+0.5\right)}\\ {}*\frac{\left(k_{3}+1\right)*qtf}{k_{3}+qtf}\;⊕\;k_{2}*nq*\frac{\left(avdl-dl\right)}{\left(avdl+dl\right)} \end{array}$$

where *N *is the number of indexed documents in the collection, *n *is the number of documents containing a specific term, *R *is the number of documents known to be relevant to a specific topic, *r *is the number of relevant documents containing the term, *tf *is within-document term frequency, *qtf *is within-query term frequency, *dl *is the length of the document, *avdl *is the average document length, *nq *is the number of query terms, the *k_i_*s are tuning constants (which depend on the database and possibly on the nature of the queries and are empirically determined), *K *equals to *k*_1 _* ((1 *- b*) + b * dl/avdl), and ⊕ indicates that its following component is added only once per document, rather than for each term.

In our experiments, the tuning constant parameters *k*_1 _and *b *are set to be different values. *k*_2 _and *k*_3 _are set to be 0 and 8 respectively. Furthermore, we have added the query expansion module on Okapi BSS, which provides two query expansion algorithms for constructing structured queries to deal with synonyms, the frequent use of acronyms and homonyms [15].

#### Indexing

One important issue that IR systems have to deal with is the size of the retrieved passages and the granularity of the indexed information. In the context of text retrieval, the granularity of the indexed text can be defined as the length of the indexed text unit and the size can be defined as the length of the retrieved passage. In this paper, we call an indexed text unit as a *passage*.

Three indices are built on the 2007 and 2006 Genomics data sets according to three passage extraction methods and a paragraph-based index is built on the 2005 and 2004 Genomics data sets [16]. A paragraph-based index is set up on the 2004 HARD data set as well. The sentence-based indexing is based on passages each of which has up to 3 sentences. The paragraph-based indexing is generated on passages each of which is a paragraph. Here a paragraph is defined as the sequence of sentences between the *<*p*>*and *<*/p*>*tags from the HTML data set. The word-based indexing forms passages using a dynamic window [16,17] .

### Experimental results

We report the baseline results in Table 1, which shows the performance under five parameter settings with three different indices in terms of the document-level, the passage-level and the passage2-level on the genomics 2004-2007 data sets and HARD 2004 data set respectively. Five groups have been set for the parameters of (*k*_1_, *b*) with their indices. Therefore, there are 15 runs on all five TREC data sets. Note that only a paragraph-based index is set up for the TREC 2005 and 2004 Genomics data sets and the TREC 2004 HARD data set.

**Table 1:** Performance of baselines

*k*_1_	b	Indices	Genomics 2007			Genomics 2006		Genomics 2005	Genomics 2004	HARD 2004	
			document	passage	passage2	document	passage	document	document	document	passage
0.4	2.0	word	0.1584	0.0675	0.0267	0.2662	0.0532	-	-	-	-
		sentence	0.1368	0.0406	0.0154	0.2378	0.0398	-	-	-	-
		paragraph	0.1086	0.0170	0.0094	0.2036	0.0192	0.1964	0.2952	0.2449	0.2635
		BEST	0.1584	0.0675	0.0267	0.2662	0.0532	0.1964	0.2952	0.2449	0.2635

0.5	1.3	word	**0.2108**	**0.0963**	0.0364	0.3140	**0.0718**	-	-	-	-
		sentence	0.1805	0.0700	0.0350	0.3030	0.0550	-	-	-	-
		paragraph	0.1588	0.0452	0.0333	0.3109	0.0369	0.2602	0.3404	0.2802	**0.2985**
		BEST	0.2108	0.0963	0.0364	0.3140	0.0718	0.2602	0.3404	0.2802	0.2985

1.0	1.0	word	0.1556	0.0434	0.0328	0.3097	0.0659	-	-	-	-
		sentence	0.1809	0.0758	0.0350	0.2918	0.0521	-	-	-	-
		paragraph	0.1902	0.0893	0.0327	0.2916	0.0337	0.2547	0.3425	0.2522	0.2718
		BEST	0.1902	0.0893	0.0350	0.3097	0.0659	0.2547	0.3425	0.2522	0.2718

1.2	0.75	word	0.1809	0.0780	0.0295	0.3045	0.0651	-	-	-	-
		sentence	0.1987	0.0814	0.0394	0.3202	0.0522	-	-	-	-
		paragraph	0.2013	0.0648	0.0578	0.3381	0.0362	**0.2874**	**0.3584**	0.2617	0.2758
		BEST	0.2013	0.0814	0.0578	0.3381	0.0651	0.2874	0.3584	0.2617	0.2758

2.0	0.4	word	0.1953	0.0844	0.0317	0.3152	0.0637	-	-	-	-
		sentence	0.2084	0.0758	0.0401	**0.3529**	0.0490	-	-	-	-
		paragraph	0.2025	0.0633	**0.0641**	0.3476	0.0362	0.2779	0.3483	**0.2810**	0.2895
		BEST	0.2084	0.0844	0.0641	0.3529	0.0637	0.2779	0.3483	0.2810	0.2895

The baseline results are presented: (1) five parameter settings for (*k*_1_, *b*) at the first and second columns; (2) three different indices, where "word" stands for the word-based index, "sentence" for the sentence-based index and "paragraph" for the paragraph-based index; (3) three evaluation measures as the document-level, the passage-level and the passage2-level; (4) five TREC data sets as the TREC 2004-2007 Genomics data sets and the TREC 2004 HARD data set; (5) only a paragraph-based index is set up for the TREC 2005 and 2004 Genomics data sets and the TREC 2004 HARD data set, as mentioned in the section of indexing.

Corresponding to the baseline results, we generate the results of the term association approach using our proposed algorithms. The performance and improvements are presented in Table 2. The values in the parentheses are the relative rates of improvement over the original results.

**Table 2:** Performance of the term association approach

*k*_1_	b	Indices	Geno 2007			Geno 2006		Geno 2005	Geno 2004	HARD 2004	
			document	passage	passage2	document	passage	document	document	document	passage
0.4	2.0	word	0.2060	0.1296	0.0526	0.2790	0.0765	-	-	-	-
			(30.06%)	(92.00%)	(96.87%)	(4.80%)	(43.87%)	-	-	-	-
		sentence	0.1710	0.0955	0.0330	0.2477	0.0698	-	-	-	-
			(25.01%)	(135.26%)	(114.11%)	(4.14%)	(75.50%)	-	-	-	-
		paragraph	0.1508	0.0726	0.0336	0.2161	0.0365	0.2156	0.3001	0.2458	0.2683
			(38.90%)	(326.77%)	(256.97%)	(6.15%)	(90.16%)	(9.78%)	(1.66%)	(0.37%)	(1.82%)

0.5	1.3	word	0.2668	**0.1611**	0.0650	0.3445	**0.1010**	-	-	-	-
			(31.31%)	(67.31%)	(78.66%)	(9.71%)	(40.64%)	-	-	-	-
		sentence	**0.2724**	0.1392	0.0619	0.3376	0.0889	-	-	-	-
			(45.38%)	(98.81%)	(76.99%)	(11.43%)	(61.59%)	-	-	-	-
		paragraph	0.1953	0.1040	0.0638	0.3270	0.0579	0.2879	0.3459	0.2843	**0.3031**
			(23.01%)	(130.18%)	(91.48%)	(5.17%)	(56.88%)	(10.65%)	(1.62%)	(1.46%)	(1.54%)

1.0	1.0	word	0.2385	0.1425	0.0526	0.3428	0.0975	-	-	-	-
			(53.28%)	(228.44%)	(60.23%)	(10.67%)	(47.89%)	-	-	-	-
		sentence	0.2251	0.1345	0.0551	0.3202	0.0842	-	-	-	-
			(24.43%)	(77.44%)	(57.29%)	(9.73%)	(61.68%)	-	-	-	-
		paragraph	0.1955	0.0969	0.0564	0.3069	0.0529	0.2777	0.3498	0.2594	0.2801
			(2.80%)	(8.50%)	(72.57%)	(5.25%)	(57.04%)	(9.03%)	(2.13%)	(2.85%)	(3.05%)

1.2	0.75	word	0.2469	0.1381	0.0547	0.3221	0.0881	-	-	-	-
			(36.49%)	(77.03%)	(84.33%)	(5.77%)	(35.28%)	-	-	-	-
		sentence	0.2698	0.1483	0.0667	0.3457	0.0823	-	-	-	-
			(35.77%)	(82.18%)	(69.35%)	(7.95%)	(57.74%)	-	-	-	-
		paragraph	0.2348	0.1155	0.0778	0.3483	0.0444	**0.3085**	**0.3606**	0.2659	0.2812
			(16.64%)	(78.31%)	(34.56%)	(3.01%)	(22.64%)	(7.34%)	(0.61%)	(1.60%)	(1.96%)

2.0	0.4	word	0.2450	0.1355	0.0568	0.3228	0.0763	-	-	-	-
			(25.44%)	(60.53%)	(79.09%)	(2.42%)	(19.71%)	-	-	-	-
		sentence	0.2605	0.1327	0.0622	**0.3549**	0.0697	-	-	-	-
			(25.01%)	(75.08%)	(55.20%)	(0.58%)	(42.27%)	-	-	-	-
		paragraph	0.2308	0.1084	**0.0762**	0.3533	0.0521	0.2889	0.3502	**0.2845**	0.2956
			(13.95%)	(71.30%)	(18.86%)	(2.78%)	(44.01%)	(3.96%)	(0.55%)	(1.25%)	(2.11%)

The results of the term association approach are presented: (1) all the runs are under the same settings as the baselines in Table 1, including the parameter settings of (*k*_1_, *b*), the indices and the evaluation measures; (2) the factor analysis base algorithms and the recursive re-ranking algorithms are applied; (3) the values in the parentheses are the relative rates of improvement over the original results.

#### Influence of parameter settings and indices

In order to investigate the influence of different indices and parameter settings, we will deeply analyse the experimental results. First, taking the TREC Genomics 2007 and 2006 data sets as an example, we compute the max, min, mean and sample standard deviation of the baselines in Table 3. From this table, we can see how these settings effect the result, since there is a disparity between the max and the min values under all the measures. Focusing on the sample standard deviation, the SSD values are calculated as a sample standard deviation of a discrete random variable. Compared to the mean, the SSD also shows the influence of the different indices and parameter settings.

**Table 3:** MAX, MIN, mean and SSD of the Genomics 2007 and 2006 baselines

	Genomics 2007			Genomics 2006	
	document	passage	passage2	document	passage
MAX	0.2108	0.0963	0.0641	0.3529	0.0718
MIN	0.1086	0.017	0.0094	0.2036	0.0192
Mean	0.1778	0.0662	0.0346	0.3005	0.0487
SSD	0.0291	0.0214	0.0136	0.0397	0.0147
	(-16.37%)	(-32.33%)	(-39.17%)	(-13.20%)	(-30.18%)

We compute the max, min, mean and sample standard deviation (SSD) of baselines on the TREC Genomics 2007 and 2006 data sets: (1) the parameter settings of (*k*_1_, *b*) and the different indices effect the baseline results greatly, since there is a disparity between the max and the min values under all the measures; (2) the SSD values are calculated as a sample standard deviation of a discrete random variable; (3) the values in the parentheses are the relative rates of improvement over the means; (4) the SSD also reflects the influence of the different indices and parameter settings of (*k*_1_, *b*).

**Figure 1:**
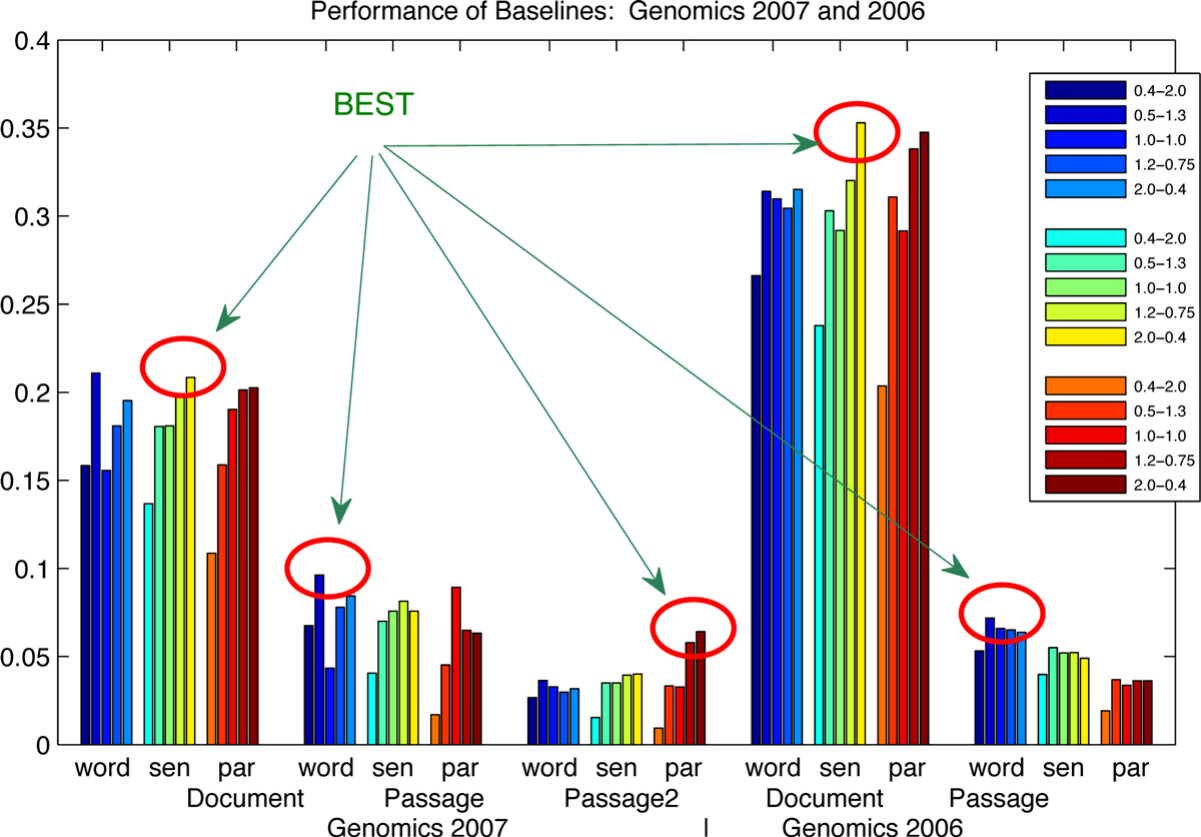
**Performance of baselines: Genomics 2007 and 2006**. The influence of index and parameter settings is investigated on the baselines: (1) the circles highlight the best results generated by three different indices; (2) the x-axis represents the evaluation measures, where "word", "sen" and "par" stand for the word-based, the sentence-based and the paragraph-based indices; the parameter settings are specified in the legend corresponding to the indices; (3) one of the conclusions is drawn that the sentence-based index produces the best results in terms of the document-level, the word-based index for the best results in terms of the passage-level and the paragraph-based index for the best results in terms of the passage2-level; (4) the data are corresponding to Table 2.

**Figure 2:**
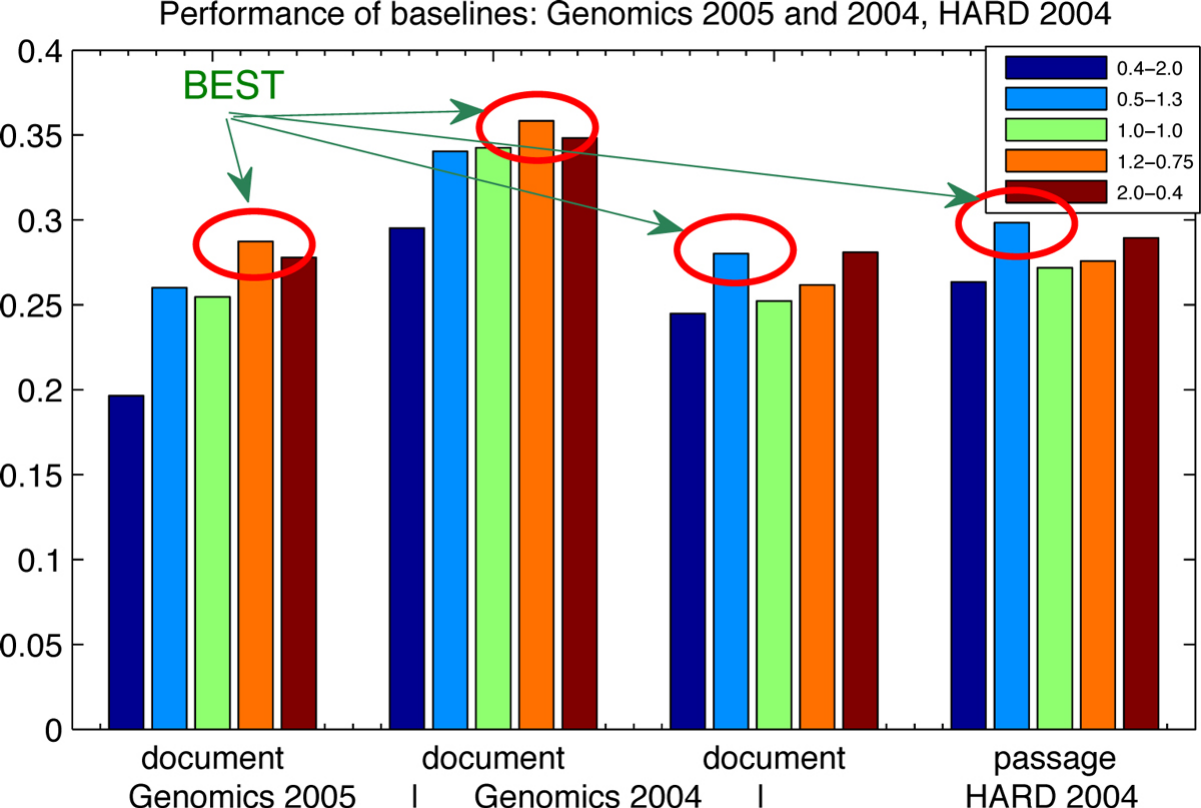
**Performance of baselines: Genomics 2005 and 2004, HARD 2004**. The influence of index and parameter settings is investigated on the baselines: (1) the circles highlight the best results; (2) only an index as the paragraph-based index, has been generated on the Genomics 2005 and 2004 data sets, the HARD 2004 data set, as mentioned in the indexing section; (3) the parameter settings are specified in the legend corresponding to the indices; (4) the data are corresponding to Table 2.

To illustrate the results in Table 1 graphically, we re-plot these data in Figure 1 and Figure 2. The performance of the baseline results is shown in terms of the document-level, the passage-level and the passage2-level. The x-axis represents the evaluation measures, where "word", "sen" and "par" stand for the word-based, the sentence-based and the paragraph-based indices. The y-axis shows the MAP performance. This figure shows that the sentence-based index produces the best results in terms of the document-level, the word-based index for the best results in terms of the passage-level and the paragraph-based index for the best results in terms of the passage2-level. This finding also confirms our motivation for building up different indices for different information needs.

#### Influence of term association

**Figure 3:**
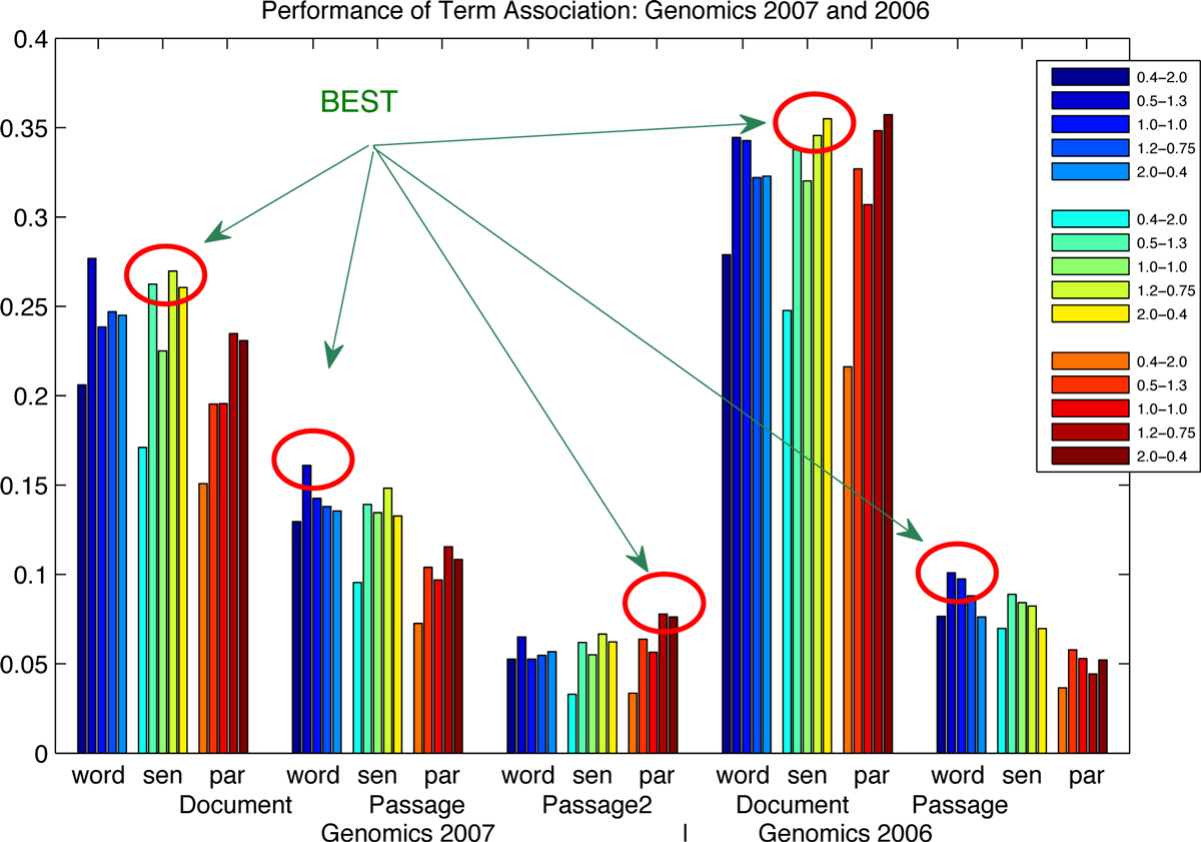
**Performance of the term association approach: Genomics 2007 and 2006**. The influence of index and parameter settings is continued on term association: (1) the circles highlight the best term association results, where the best term association results come from the best baselines; (2) the same index finding can also be observed that the sentence-based index produces the best term association results in terms of the document-level, the word-based index for the best in terms of the passage-level and the paragraph-based index for the best in terms of the passage2-level; (3) the data are corresponding to Table 3.

**Figure 4:**
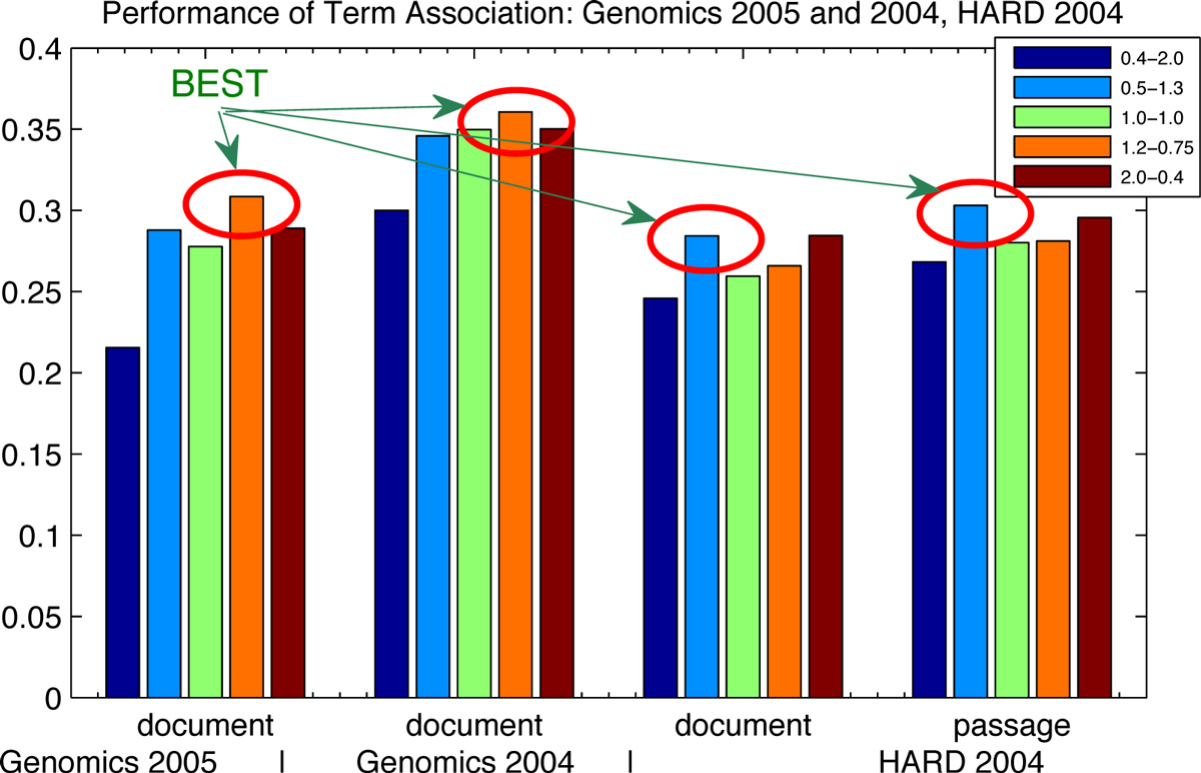
**Performance of the term association approach: Genomics 2005 and 2004, HARD 2004**. The influence of index and parameter settings is continued on term association: (1) the circles highlight the best term association results, where the best term association results come from the best baselines; (2) the data are corresponding to Table 3.

In order to illustrate the term association results in Table 2, we plot them graphically in Figure 3 and Figure 4. It clearly shows that, for all the measures on five TREC data sets, the term association approach always outperforms the baselines. The improvements in the parentheses explain the significance evidently. More interesting, the figures of the factor analysis results almost have the same distributions as the figures of baselines. The best factor analysis results always come from the best baseline results. The sentence-based index produces the best factor analysis results in terms of the document-level, the word-based index for the best factor analysis results in terms of the passage-level and the paragraph-based index for the best factor analysis results in terms of the passage2-level.

**Figure 5:**
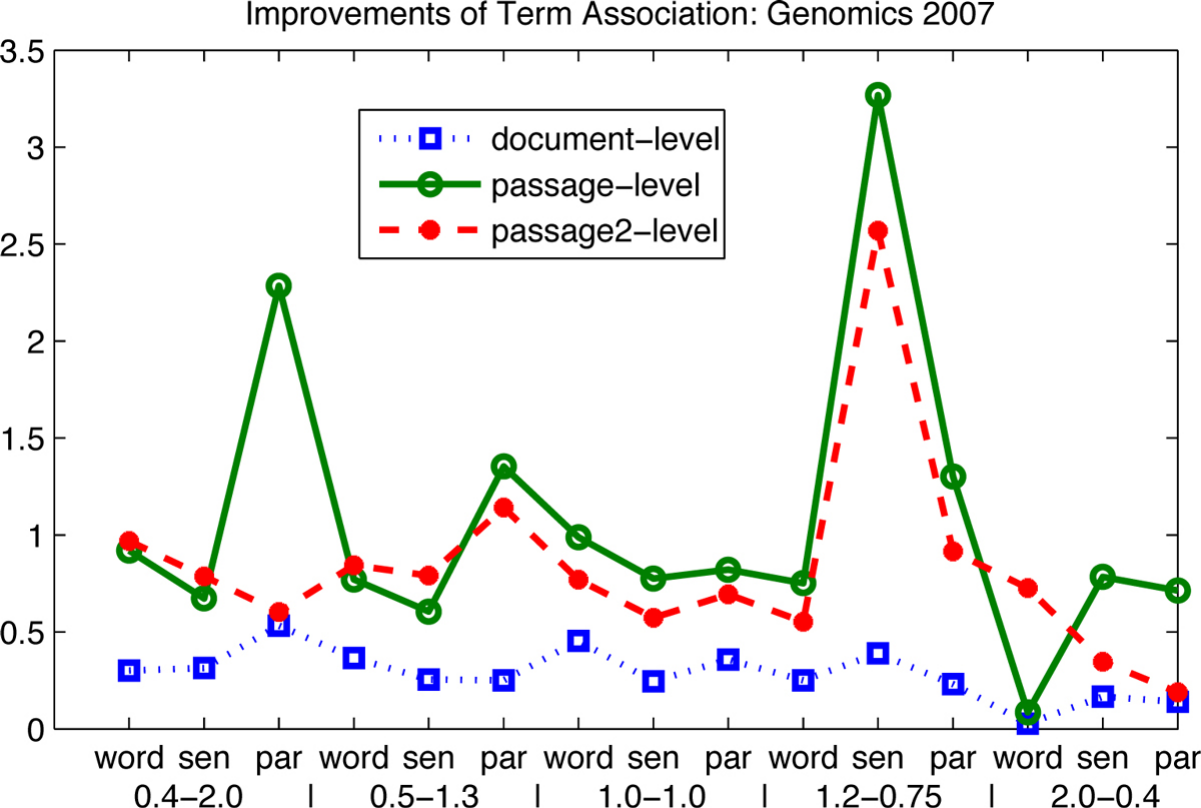
**Improvements of the term association approach over baselines: Genomics 2007**. The improvements of term association over baselines are investigated: (1) the proposed approach outperforms the baselines, since the lines are in the first quadrant; (2) the influence on the passage levels is greater than that on the document-level; (3) the data are corresponding to Table 3.

**Figure 6:**
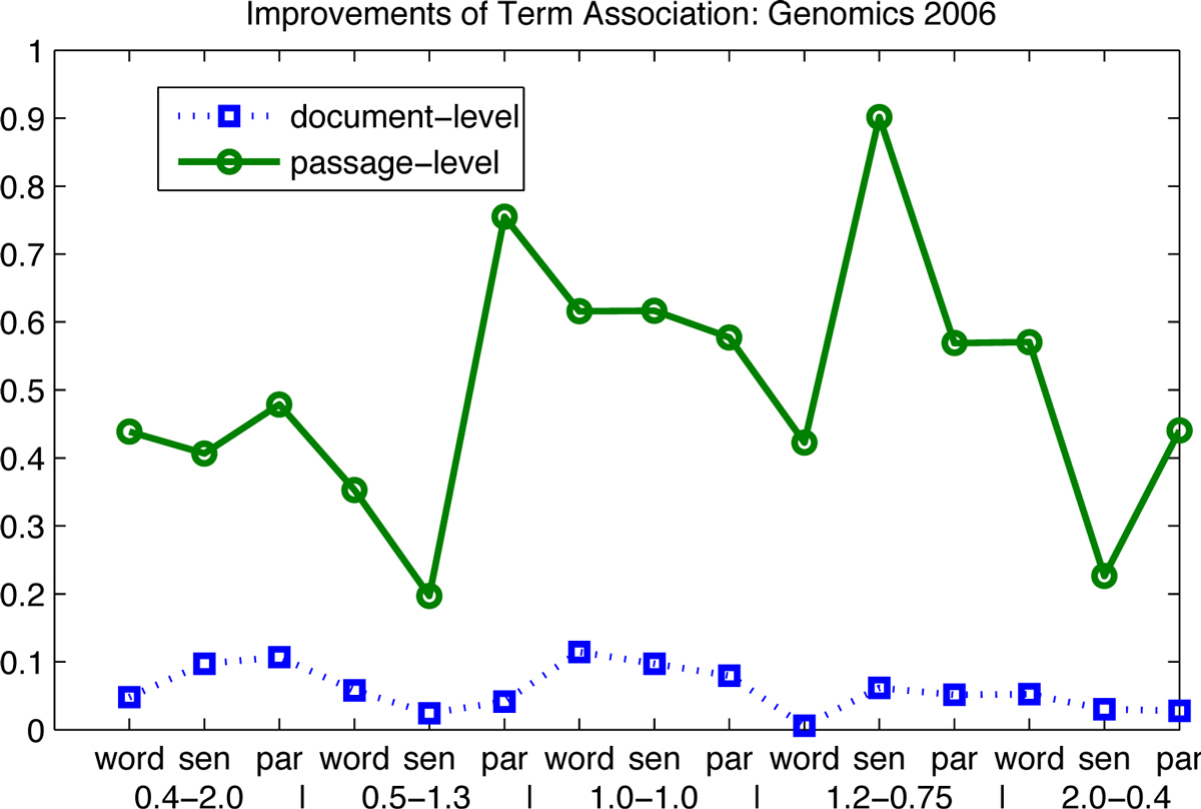
**Improvements of the term association approach over baselines: Genomics 2006**. The improvements of term association over baselines are investigated: (1) the proposed approach outperforms the baselines, since the lines are in the first quadrant; (2) the influence on the passage levels is greater than that on the document-level; (3) the data are corresponding to Table 3.

**Figure 7:**
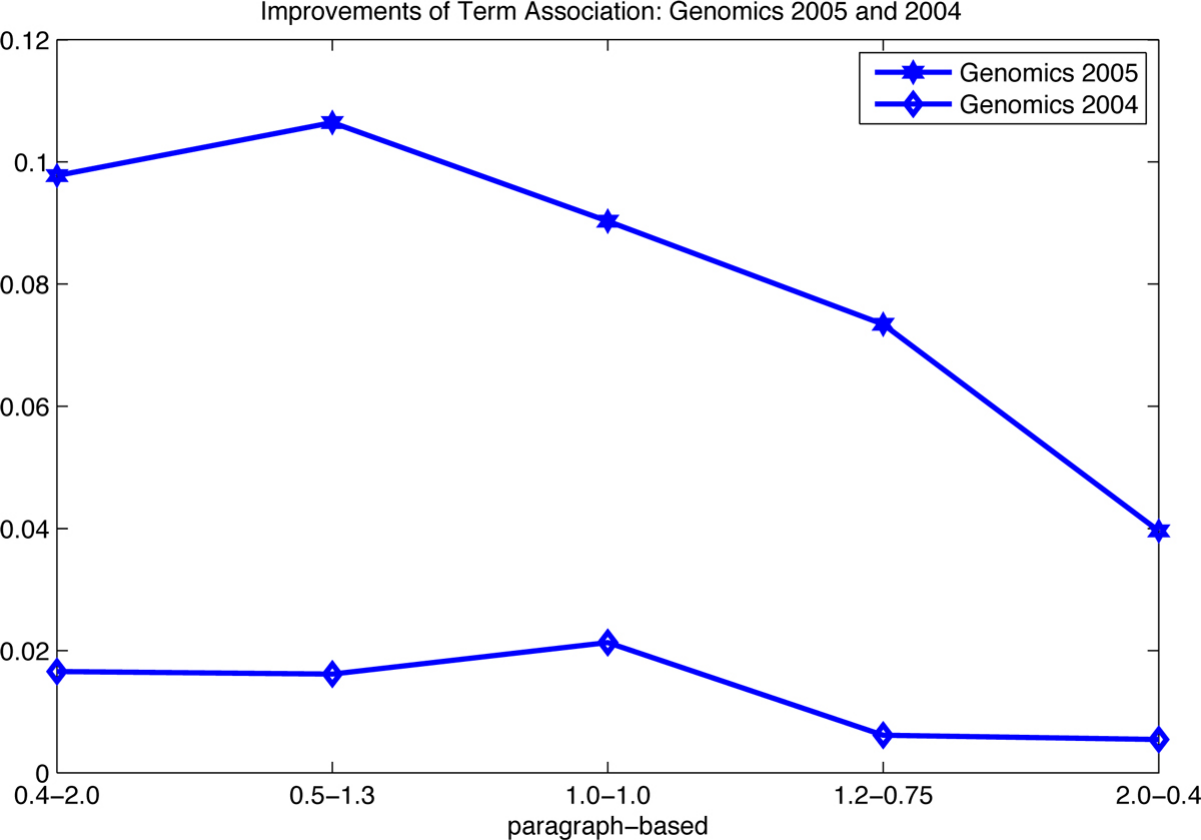
**Improvements of the term association approach over baselines: Genomics 2005 and 2004**. The improvements of term association over baselines are investigated: (1) the proposed approach outperforms the baselines, since the lines are in the first quadrant; (2) no passage level improvement lines on the Genomics 2005 and 2004 data sets are presented, since there is only the document-level; (3) the data are corresponding to Table 3.

**Figure 8:**
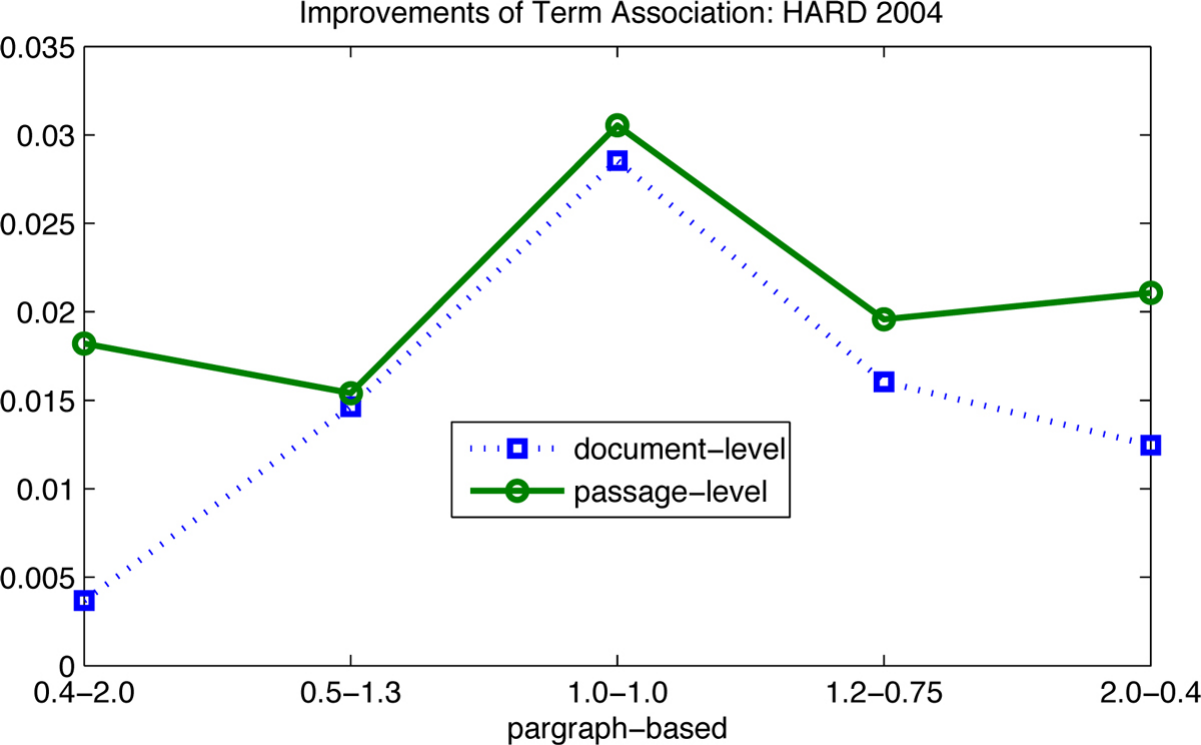
**Improvements of the term association approach over baselines: HARD 2004**. The improvements of term association over baselines are investigated: (1) the proposed approach outperforms the baselines, since all the lines are in the first quadrant; (2) the influence on the passage levels is greater than that on the document-level; (3) the data are corresponding to Table 3.

In order to illustrate the improvements of term association, in Table 2, we plot them graphically in Figure 5, Figure 6, Figure 7 and Figure 8. There are two observations as follows. First, the positive values of the improvements notify that term association carries important weight on the retrieval results, which is much better than the baselines that only consider the unigram keywords independently. In other words, those bigram and trigram associations have more influential in the retrieval results than the independent keywords. Second, the influence in terms of the passage levels (the passage2-level and the passage-level) is greater than that in terms of the document-level. We also can see in Figure 5, Figure 6 and Figure 8, that the absolute values of improvements on the passage-level are much higher than those on the document-level. This can be explained that term association is more efficient to be applied in the sentences or paragraphs compared to the documents.

#### Influence of K for recursive re-ranking

We initialize the depth as *k *= 10 in the recursive re-ranking algorithm. The number *k *stands for the top *k *term associations weighted by the factor analysis based model. We recursively re-rank the retrieved passages according to whether the passages contain the top *k *term associations or not. We conduct a series of experiments with different settings of *k *values in order to investigate the influence of value *k *and find a local optimization value for the proposed algorithm. We first randomly choose five original baselines from our five data sets respectively, namely Genomics 2007, Genomics 2006, Genomics 2005, Genomics 2004 and HARD 2004. Then the factor analysis model is applied on the baselines. Five numbers such as 1, 5, 10, 20, 100, are tested and the performance is shown in Table 4. We can see that number *k *affects the performance greatly when *k *is smaller than 10. However, when *k *becomes larger than 10, the final performance almost has no change. Therefore, we get this local optimization number as 10 for *k *in the recursive re-ranking algorithm for all the runs.

**Table 4:** Number k discussion

	*n*	document	passage	passage2
Genomics 2007	1	0.3012	0.0918	0.1436
	5	0.3349	0.1400	0.1588
	10	0.3438	0.1422	0.1635
	20	0.3438	0.1422	0.1635
	100	0.3438	0.1422	0.1635

Genomics 2006	1	0.3974	0.1401	-
	5	0.4049	0.1445	-
	10	0.4087	0.1467	-
	20	0.4083	0.1466	-
	100	0.4083	0.1466	-

Genomics 2005	1	0.3012	-	-
	5	0.3116	-	-
	10	0.3123	-	-
	20	0.3123	-	-
	100	0.3123	-	-

Genomics 2004	1	0.3470	-	-
	5	0.3555	-	-
	10	0.3584	-	-
	20	0.3584	-	-
	100	0.3584	-	-

HARD 2004	1	0.2015	0.2005	-
	5	0.2223	0.2197	-
	10	0.2250	0.2208	-
	20	0.2248	0.2208	-
	100	0.2248	0.2208	-

The number **k **is the parameter in the recursive re-ranking algorithm: (1) the empirical study makes a local optimization number *k *= 10 as the final depth in the final experiments; (2) *k *stands for the top *k *term associations weighted by the factor analysis based model; (3) the recursive re-ranking algorithm will re-rank the baselines according to these *k *terms; (4) the more the results contain terms among these *k *terms, the higher ranking scores the results obtain; (5) five numbers such as 1, 5, 10, 20, 100, are tested; (6) five original baselines from our five data sets respectively, namely Genomics 2007, Genomics 2006, Genomics 2005, Genomics 2004 and HARD 2004; (7) *k *affects the performance greatly when *k *is smaller than 10, while the final performance almost has no change if *k *becomes larger than 10.

#### Comparison with GSP algorithm

We adopt the GSP algorithm as a comparison to our proposed approach. In order to map the GSP algorithm to our research problem, we treat the keywords extracted from the queries as the singleton items and *N *passages retrieved by the system for each query as the transaction database. Therefore, the candidates of 1 *- sequences *are all the keywords, the *k - sequences *candidates are generated on the frequent (*k - *1) - *sequences*. For the support counting, we define the minimum support value corresponding to each query as follows. First, the counts of candidates are automatically calculated by the modified GSP algorithm, including all *k - sequences*. Then, we simulate the counts as a non-parametric distribution. Third, the 95% confidence interval of this distribution is computed, where the lower bound is the minimum support value for this GSP algorithm.

In this section, we study how the GSP algorithm performs on our five data sets. Here we focus on the experimental results with the paragraph index under five parameter settings, as shown in Table 5.

**Table 5:** Performance of GSP algorithm

	(*k*_1_, *b*)	Geno 2007			Geno 2006		Geno 2005	Geno 2004	HARD 2004	
		document	passage	passage2	document	passage	document	document	document	passage
GSP	(0.4,2.0)	0.1066	0.0338	0.0149	0.1892	0.0242	0.1867	0.2723	0.2358	0.2639
		(-1.87%)	(-98.75%)	(-58.28%)	(-7.09%)	(-25.95%)	(-4.96%)	(-7.74%)	(-3.72%)	(-0.15%)
	(0.5,1.3)	0.149	0.0843	0.0456	0.2855	0.0466	0.2423	0.3165	0.2562	0.3001
		(-6.18%)	(-86.59%)	(-36.85%)	(-8.17%)	(-26.31%)	(-6.88%)	(-7.01%)	(-8.57%)	(-0.54%)
	(1.0,1.0)	0.1839	0.0898	0.0357	0.2757	0.0402	0.2385	0.3166	0.2501	0.2842
		(-3.32%)	(-0.60%)	(-9.21%)	(-5.46%)	(-19.40%)	(-6.36%)	(-7.55%)	(-0.83%)	(-4.56%)
	(1.2,0.75)	0.1905	0.0714	0.0658	0.3174	0.0404	0.2655	0.3293	0.2589	0.2776
		(-5.35%)	(-10.11%)	(-13.79%)	(-6.11%)	(-11.65%)	(-7.62%)	(-8.11%)	(-1.07%)	(-0.65%)
	(2.0,0.4)	0.1931	0.0657	0.0667	0.3203	0.0403	0.2588	0.3206	0.2567	0.2916
		(-4.62%)	(-3.79%)	(-4.02%)	(-7.85%)	(-11.40%)	(-6.89%)	(-7.96%)	(-8.65%)	(-0.73%)
	Best	0.1931	0.0898	0.0667	0.3203	0.0466	0.2655	0.3293	0.2589	0.3001

Baselines	Best	0.2108	0.0963	0.0641	0.3529	0.0718	0.2874	0.3584	0.281	0.2985

TA	Best	0.2724	0.1611	0.0762	0.3549	0.101	0.3085	0.3606	0.2845	0.3031

The GSP algorithm is adopted as a comparison to the proposed approach: (1) the candidates of 1 - *sequences *are all the keywords, the *k *- *sequences *candidates are generated on the frequent (*k *- 1) - *sequences*, after mapped the GSP algorithm to our research problem; (2) the counts of candidates are simulated as a non-parametric distribution, where the lower bound of the 95% confidence interval is the minimum support value for this GSP algorithm; (3) only the paragraph index under five parameter settings of (*k*_1_, *b*) is considered; (4) the best results of the GSP algorithm are compared with the best of the baselines and the proposed term association approach; (5) "TA" stands for term association; (6) the values in the parentheses are the relative rates of improvement over the original baselines.

Furthermore, we compare the best results of the GSP algorithm, the baselines and the proposed term association approach.

An interesting finding is drawn from the results of the GSP algorithm. The GSP algorithm works very well in terms of the passage-level and the passage2-level, while it is not good for the document-level. This can be explained by the following scenario. The frequent 3 - *sequence T*_1_*T*_3_*T*_4 _is found in the documents *D*_1 _and *D*_2_. In *D*1, *T*_1_*T*_3_*T*_4 _is contained in a short passage so that *D*_1 _earns good MAP results on the document-level and the passage-level. In the document *D*_2_, the situation is that *T*_1_, *T*_3 _and *T*_4 _are found in different passages respectively. Since *T*_1_*T*_3_*T*_4 _is still found as a sequence based on the definitions, *D*_2 _is given a high weight and is going to earn good performance at least on the document-level. However, the standard evaluation does not think *D*_2 _is qualified to be a relative document so that *D*_2 _decreases the performance of the document-level.

Compared to the GSP algorithm, the proposed term association approach outperforms the baselines and the GSP results on all the measures. The factor analysis based model considers not only the concurrence of the terms, but also the dependency, especially in the high order structure. In the GSP algorithm, the document *D*_2 _is given a good score. However, in the factor analysis based model, the factor loadings of *T*_1_*T*_3_*T*_4 _in *D*_2 _is very small, since *T*_1_*T*_3_*T*_4 _is not treated as a trigram term association. *T*_1_, *T*_3 _and *T*_4 _are three unigram terms, while *T*_1_*T*_3_*T*_4 _is a frequent 3 - *sequence *in the GSP algorithm. So the proposed approach avoids assigning a high weight to the document *D*_2_.

The major difference among our proposed approach, ngram and PLSA, is that term associations are not dependent on the previous associations, whose reliance and importance are decided by the dependencies among the keywords in the passages, not by their probabilities upon the previous terms. For example, an interesting finding using factor analysis in this work, is that the bigram *k*_1_*k_j_*(*j *≠ 1) might have the highest reliance, even though their previous unigram term *k*_1 _or *k_j _*is not the most important for a query in some IR systems. And our experiment confirms that *k*_1_*k_j _*plays an important role in the improved re-ranking result. Therefore, one of the major contributions of the proposed approach is to extract subsequences as term associations from a query without preliminary knowledge. This promotes us to employ the GSP algorithm as a comparison to evaluate the proposed approach statistically, but not to compare this approach with PLSA and PCA.

#### Comparison with official submissions

In order to further evaluate the term association approach to improving performance, we compare the performance of the term association approach to the official submissions at the best and mean values on the five TREC data sets in Table 6. Since the submissions of the 2004 HARD data set are not officially released, we focus on the genomics data sets. We can observe that, for the mean performance, term association outperforms baselines and the official submissions. For some best performance, term association makes improvements on baselines, but is not as good as the official submissions. However, based on the discussion upon the influence of term association in the section of influence of term association, we believe we could achieve higher performance if we have better baselines.

**Table 6:** Comparisons of baselines, term associations and official submissions

		Geno 2007			Geno 2006		Geno 2005	Geno 2004	HARD 2004	
		document	passage	passage2	document	passage	document	document	document	passage
Baselines	Best	0.2108	0.0963	0.0641	0.3529	0.0718	0.2874	0.3584	0.2810	0.2985
	Mean	0.1778	0.0662	0.0346	0.3005	0.0487	0.2553	0.3370	0.2640	0.2798

TA	Best	0.2724	0.1611	0.0762	0.3549	0.1010	0.3085	0.3606	0.2845	0.3031
	Mean	0.2273	0.1236	0.0579	0.3182	0.0719	0.2757	0.3413	0.2680	0.2857

Official	Best	0.3105	0.0976	0.1097	0.5439	0.1486	0.3020	0.4075	-	-
	Mean	0.1891	0.0582	0.0421	0.2887	0.0392	0.1968	0.2074	-	-

The performance of the proposed term association approach is compared to the official submissions: (1) all the results are compared at the best values and the mean values; (2) the submissions of the TREC 2004 HARD data set are not officially released; (3) "TA" stands for term association and "official" for official submissions.

#### A case study

Topic 200 of the TREC 2007 queries is taken as an example. The description for Topic 200 is "What serum [PROTEINS] change expression in association with high disease activity in lupus?". Nine keywords are extracted as serum, proteins, change, expression, association, high, disease, activity and lupus. The rest words are removed by the system as the stop words. The system stems the keywords as serum, protein, chang, express, associ, high, diseas, active and lupus.

Table 7 shows the baseline whose parameters are set as (*k*_1_, *b*) = (2.0, 0.4) with the paragraph-based index. The information of its keywords, the term count, the frequency and rank are presented for Topic 200. The parameters for this baseline are (*k*_1_, *b*) = (2.0, 0.4) with the paragraph-based index. There are totally $\left(C_{9}^{1}+C_{9}^{2}+C_{9}^{3}\right)$term associations generated by the proposed approach. Table 8 presents the top 10 term associations after applying the factor analysis based model, where terms, term count and their communalities are presented. Then in Table 9, the performance of term association is compared with the performance of baseline of Topic 200 in terms of the document-level, the passage-level and the passage2-level.

**Table 7:** Topic 200: keyword frequency rank

#	Term	Term count	Percentage	Rank
1	Lupus	869	23.80%	1
2	Diseas	753	20.70%	2
3	Activ	496	13.60%	3
4	Associ	476	13.10%	4
5	Serum	294	8.10%	5
6	High	274	7.50%	6
7	Protein	195	5.40%	7
8	Express	179	4.90%	8
9	Chang	108	3.00%	9

The original information of the keywords for Topic 200 is shown: (1) terms are extracted with stemming; (2) term counts are obtained from the first round retrieved passages, which are the top 1000 retrieved passages as the baseline; (3) the percentage is calculated based on 9 terms; (4) the rank depends on the term counts; (4) the parameters for this baseline are (*k*_1_, *b*) = (2.0, 0.4) with the paragraph-based index.

**Table 8:** Topic 200: ranking term associations

Rank	Term association	Term count	Communalities
1	high lupus serum	33	69.4
2	lupus protein serum	47	62.7
3	activ lupus serum	7	61.2
4	activ serum	118	60.0
5	activ associ diseas	124	59.9
6	associ diseas lupus	162	59.5
7	activ associ high	7	59.2
8	lupus serum	116	58.5
9	diseas high lupus	90	58.0
10	associ protein	20	58.0

Term associations are presented by applying the factor analysis based model: (1) the top 10 term associations are shown among $\left(C_{9}^{1}+C_{9}^{2}+C_{9}^{3}\right)$ term associations generated by the proposed approach; (2) term count are computed from the top 1000 documents of the baseline; (3) the communality of each term association is calculated by the factor analysis based model; (4) the rank of term associations is given by their communalities.

**Table 9:** Topic 200: performance comparison

	document	passage	passage2
Baseline	0.3752	0.1546	0.0688
Term association	0.4238	0.2157	0.0811
Improvements	(12.95%)	(39.52%)	(17.88%)

The performance of term association is compared with the performance of baseline of Topic 200 in terms of the document-level, the passage-level and the passage2-level: (1) the values in the parentheses are the relative rates of improvement over the baselines; (2) term association outperforms baseline.

First of all, we can see that no unigram is in the ranking association list. All the term associations in Table 8 are bigrams and trigrams. Since the term association improved result outperforms the baseline, it means that term association works very well on all the measures. Therefore, term association is better than only considering the keywords independently. Second, the trigram "high lupus serum" has the higher reliance than the bigram "activ serum", although the trigram's term count is only 7, which is much less than the bigram's term count as 118. This tells us that the term frequency might not make sense when compared to term association.

## Conclusions

Modelling term association for improving biomedical information retrieval using factor analysis, is one of the major contributions of our work. We investigate term association among the keywords from a query and then build up a factor analysis based model to investigate the significance of term association. The proposed approach works very well on five large TREC data sets. Our improved performance is among top TREC official results submitted in the TREC 2004-2007 Genomics data sets and the TREC 2004 HARD data set.

Term association considering co-occurrence and dependency among the keywords produces better results than the baselines treating the keywords independently. In the other hand, the unigrams, bigrams and trigrams are terms independently computed by the factor analysis based model, which means that the trigrams are not dependent on the bigrams' importance, and the bigrams are not dependent on the unigrams' importance. Their importance is decided by the model and the appearances in the passages. This is also confirmed by the GSP algorithm.

In the term association approach, keywords and the retrieved passages are the observable data, and the factor analysis based model is built up to discover the unobservable latent factors. Factor loadings are computed to indicate the weights of the common factors. Communalities are calculated based on factor loadings to represent the importance and reliance of the corresponding terms associations. Finally, a ranking term association list is given by the model. Then we recursively re-rank the baselines and report the experimental results.

The experimental results show that term association outperforms the baselines and the GSP results on all the evaluation measures, which provides a promising avenue for improving the information retrieval performance. Our future work includes investigating the PLSA model on the genomics research. This is also our ongoing work.

## Methods

We will first introduce the observations. Then a factor analysis based model is proposed, in which common factors, factor loadings and communalities are defined. The pseudo codes for the factor analysis based algorithm and the recursive re-ranking algorithm are shown respectively.

### Observations

In the traditional IR systems, keywords extracted from the queries are used to retrieve documents/passages with some weighting functions. In this paper, we examine term associations among keywords to improve information retrieval performance. For example, there are *n *keywords extracted from a query, and the system gives *N *passages for each retrieval baseline result. Term associations among these *n *keywords are extracted and used for re-ranking the *N *passages.

Our two main observation files from the system are: 1) the baseline result retrieved by the system with *N *passages for each query; 2) the corresponding term file which displays how many and which keywords are retrieved in each passage. The sample data are presented in Table 10 and 11.

**Table 10:** Sample of retrieval passage list

Topic #	Document ID	Rank	Weight	Offset	Length	Label
200	12595615	1	48.63	28426	295	yorkuga1
200	12595615	2	46.25	3839	339	yorkuga1
200	15814577	3	43.338	5656	125	yorkuga1
...	...	...	...	...	...	...

The baseline format is presented: (1) the top 1000 passages are shown for each topic; (2) each document contains multiple passages such that some retrieved passages share the same document ID; (3) the rank is given by the weight; (3) the weights are given by the IR system; (4) the offset is the starting point of the passage, and the length is the passage length which removes the stopping words; (5) the label is to identify the specific run.

**Table 11:** Sample of the corresponding term file

Topic #200
passage #1: 4 of the 9 terms was found *>>*activ associ diseas lupus
passage #2: 4 of the 9 terms was found *>>*activ associ diseas lupus
passage #3: 3 of the 9 terms was found *>>*activ diseas lupus
... ...

The term file displays how many and which keywords are retrieved: (1) the numbers are those terms retrieved in a passage, instead of a document; (2) the term file is a temporary file generated by our IR system; (3) the passage number is corresponded to the rank of the baseline.

Taking *n *keywords as a sequence, we study 1-keyword subsequence, 2-keyword subsequence and 3-keyword subsequence as unigram, bigram and trigram term associations. If one term is appeared in a passage, it scores 1; if not, it scores 0. Therefore each passage can be presented as a 1-0 vector as shown in Table 12.

**Table 12:** Observation of keyword associations

#	unigram			bigram			trigram		
	*T*_1_	**.**.	*T_n_*	*T_n_*_+1_	**.**.	^$T_{C_{n}^{1}+C_{n}^{2}}$^	^${T_{C_{n}^{1}}}_{+C_{n}^{2}+1}$^	**.**.	^$T_{C_{n}^{1}+C_{n}^{2}+C_{n}^{3}}$^
	*k*_1_	**.**.	*k_n_*	*k*_1_*k*_2_	**.**.	*k_n_*_-1_*k_n_*	*k*_1_*k*_2_*k*_3_	**.**.	*k_n_*_-2_*k_n_*_-1_*k_n_*
1	1	..	1	0	..	1	0	..	1
2	1	..	1	1	..	1	1	..	1
..		.	.		.	.		.	.
*N*	0	..	0	0	..	1	0	..	1

The keyword associations are observed: (1) 1-keyword subsequences are unigrams, 2-keyword subsequences are bigrams, 3-keyword subsequences are trigrams and so on; (2) all unigrams, bigrams, trigrams and ngrams are defined as terms; (3) a passage scores 1 if a term is appeared in it, otherwise it scores 0; (4) a passage is represented as a 1-0 vector.

### A factor analysis based model

Factor analysis is a method for investigating whether a number of variables of interest *T*_1_, *T*_2_, . . . , *T_n_*, are linearly related to a smaller number of unobservable factors *F*_1_, *F*_2_, . . . , *F_m_*.

Based on the observation data, we suggest that the observations are functions of a number of common underlying factors. The underlying factors, tentatively and rather loosely describe the unobservable features of the retrieval passages. The score over all term associations is the sum of a constant times a common factor, i.e., it is a linear combination of those common factors in Equation 2.

(2) $$\overset{m}{\underset{i=1}{\sum }}\ell_{i}\times Common\;Factor_{i}$$

where *m *stands for the count of common factors, *m ≤ n*. The numbers ℓ_1_, . . . , ℓ*_m _*are the factor loadings associated with this term association.

In this paper, term associations contain unigrams, bigrams and trigrams. Then, the data applied by the factor analysis based model would be $C_{n}^{1}+C_{n}^{2}+C_{n}^{3}$associations and *N *passages for each query, which is a $\left(C_{n}^{1}+C_{n}^{2}+C_{n}^{3}\right)\times N$matrix. The factor loadings and the common factors for each query must be inferred from the data. Here we use *n*' to denote $C_{n}^{1}+C_{n}^{2}+C_{n}^{3}.$

In order to compute the reliance of the associations, communality is defined for the *n*' associations as

(3) $$h_{i}^{2}=\ell_{i,0}^{2}+\ell_{i,1}^{2}+..+\ell_{i,m}^{2}$$

The larger of the communalities $h_{i}^{2}$ are, the more important of common factors are to represent the keywords.

It is assumed that each term association is related to *m *factors. Therefore, the mathematical model for the above example can be written as follows.

(4) $$\left\{\begin{array}{cc} T_{1} & =\ell_{1,0}+\ell_{1,1}f_{1}+..+\ell_{1,m}f_{m}+\varepsilon_{1}\\ T_{k} & \overset{...}{=}\ell_{k,0}+\ell_{kN,1}f_{1}+..+\ell_{k,m}f_{m}+\varepsilon_{k}\\ T_{n^{\prime }} & \overset{...}{=}\ell_{n^{\prime },0}+\ell_{n^{\prime },1}f_{1}+..+\ell_{n^{\prime }{,}_{m}}f_{m}+\varepsilon_{n^{\prime }} \end{array}\right)$$

where *T_k _*is the score of the *k^th ^*term association, with *k *= 1, . . . , *n*'; *< f*_1_, . . . , *f_m _>*is the unobserved common factor vector for the *k^th ^*term association; *<*ℓ*_k_*,_0_, ℓ*_k_*,_1_, . . . , ℓ*_k,m _>*are the factor loading vector of the *k^th ^*term association; *ε_k _*is error term, which serves to indicate that the hypothesized relationships are not exact. In matrix notation, we have

(5) T=LF+ε

where **T **is a *n' *× *N *matrix of observable data; **L **is a *n' *× (*m *+ 1) matrix of factor loadings, which are unobservable constants; **F **is a *n' *× *m *matrix of unobservable common factors; *ε *is a *n' × N *matrix of unobservable error variables.

Observe that by doubling the scale on which *f*_1 _of **F **is measured, and simultaneously halving the factor loadings for *f_j_*(*j *= 2..*m*) makes no differences to the model. Thus, no generality is lost by assuming that the standard deviation of *f_j_*(*j *= 2*..m*) is 1. Likewise for *f*_1_. Moreover, for similar reasons, no generality is lost by assuming every two factors *f_i _*and *f_j _*(*i *≠ *j*) are uncorrelated with each other. The "errors" *ε *are taken to be independent of each other. The variances of the "errors" associated with the *n' *different associations are not assumed to be equal. The values of the factor loadings **L **and the variances of the "errors" *ε *can be estimated given the observed data **T**.

### A factor analysis based algorithm

We present the proposed factor analysis based algorithm as follows, in which eight phases are included. The phase of **Initialization **gives the initial values for this algorithm, such as *N *= 1000. The phase of **Matrices generation **creates the matrices of the associations. In this research, we only consider unigrams, bigrams and trigrams. We calculate the communalities for all the associations at the phase of **Communality calculation**. Finally, the phase of **Re-ranking **is using the recursive re-ranking algorithm proposed in the following section of the recursive re-ranking algorithm to re-ranking the original result.

*begin*

   **0. Input**

      The baseline result for the queries on each data set.

      The term file corresponding to the baseline result.

   **1. Output**

      A re-ranking result for the queries on each data set.

   **2. Initialization**

      *N *= 1000;

      *k *= 10;

   **3. Keyword extraction**

      Read the term file;

         For each query {

            Get the keyword sequence;

            Get the value of *n*;}

   **4. Matrices generation**

      For each query {

         For (*i *= 1; *i <*= 3; *i *+ +) {

            Generate the i-keyword subsequences;

            For all the i-keyword subsequences {

               Search the subsequence in the term file;

                  If the subsequence exits, it scores 1; Else it scores 0;}}

         $\text{A}\;\left(C_{n}^{1}+C_{n}^{2}+C_{n}^{3}\right)\times N$matrix is generated. }

   **5. Communality calculation**

      For each query {

         Mathematically set up the factor analysis model;

         Estimate the factor loadings for the common factors;

         Compute the communalities; }

      Sort the associations according to their communalities;

      Get top *k *associations as the ranking association list.

   **6. Re-ranking**

      For each query {

         Call the recursive re-ranking algorithm presented

         in the following section of the recursive re-ranking algorithm; }

   **7. Final result generation**

      A final re-ranking result is generated.

*end*

First, Keywords are directly extracted from the queries. There is a term file which displays how many and which keyword terms are retrieved for each passage by the system. In other words, all the retrieved passages can be labelled by the keywords. Furthermore, for the keywords in the queries, no query expansion but stemming is applied. For example, "change" can have several expressions such as "changeless", "changing", "changeable", and so on. So our system deals with "change" as "chang". The process is done automatically in the system [13,15].

Second, according to the keyword sequence, unigrams, bigrams and trigrams are generated as term associations for each query, which makes a $C_{n}^{1}+C_{n}^{2}+C_{n}^{3}\times N$matrix.

Third, we set up the factor analysis model after generating the matrices. Through sorting the communalities, we can find which term association is more important according to its communality. The larger the communalities are, the more important the corresponding associations are. Finally, we recursively re-rank the passages as the output result using the recursive re-ranking algorithm introduced in the following section.

### A recursive re-ranking algorithm

A recursive re-ranking algorithm is called for the phase of **Re-ranking **in the previous factor analysis based algorithm. Here we present the pseudo codes as follows.

*begin*

   **0. Input**

      The baseline result for the queries on each data set.

      The ranking association list generated by the factor analysis model.

   **1. Output**

      A re-ranking passage list for the queries on each data set.

   **2. Initialization**

   *k *= 10 which will be discussed in the section of influence of K for recursive re-ranking;

   **3. Recursive division**

      For the 1st association $T_{1}^{\prime }$ in the ranking association list,

         The result list is divided into 2 parts: $P_{1}^{1}$and $P_{2}^{1},$where $P_{1}^{1}$contains $T_{1}^{\prime }$and $P_{2}^{1}$ does not.

         For each part, the passages are sorted by their given weights.

      For the 2nd association $T_{2}^{\prime }$ in the list,

             The result list is divided into 4 parts: $P_{1}^{2},P_{2}^{2}$and $P_{3}^{2},P_{4}^{2},$

            where $P_{1}^{2}$and $P_{3}^{2},$contain $T_{2}^{\prime },$while $P_{2}^{2},$and $P_{4}^{2}$do not.

        Repeat to re-rank the result list for *k *times.

            The result list is divided into 2*^k ^*parts.

            The odd parts contain the associations while the even parts do not.

   **4. Re-ranking**

        For (*i *= 1; *i *≤ 2*^k^*; *i *+ +) {

        Sort the passages in $P_{i}^{k}$according to their weights;

            *q*(*i*) = # of $P_{i}^{k}$}

        Let *RL *is the final re-ranking list, *v *is the size of *RL*;

        The first *q*(1) passages in *RL *are the passages in $P_{1}^{k};$

        For $P_{i+1}^{k}\{$

            *v*(*i*) = # of *RL*;

            The passages from (*v*(*i*) + 1) to (*v*(*i*) + *q*(*i *+ 1)) in *RL *are the passages in $P_{i+1}^{k};$

            *i *= *i *+ 1; }

   **5. Final result generation**

      A re-ranking result list is generated.

*end*

**Figure 9:**
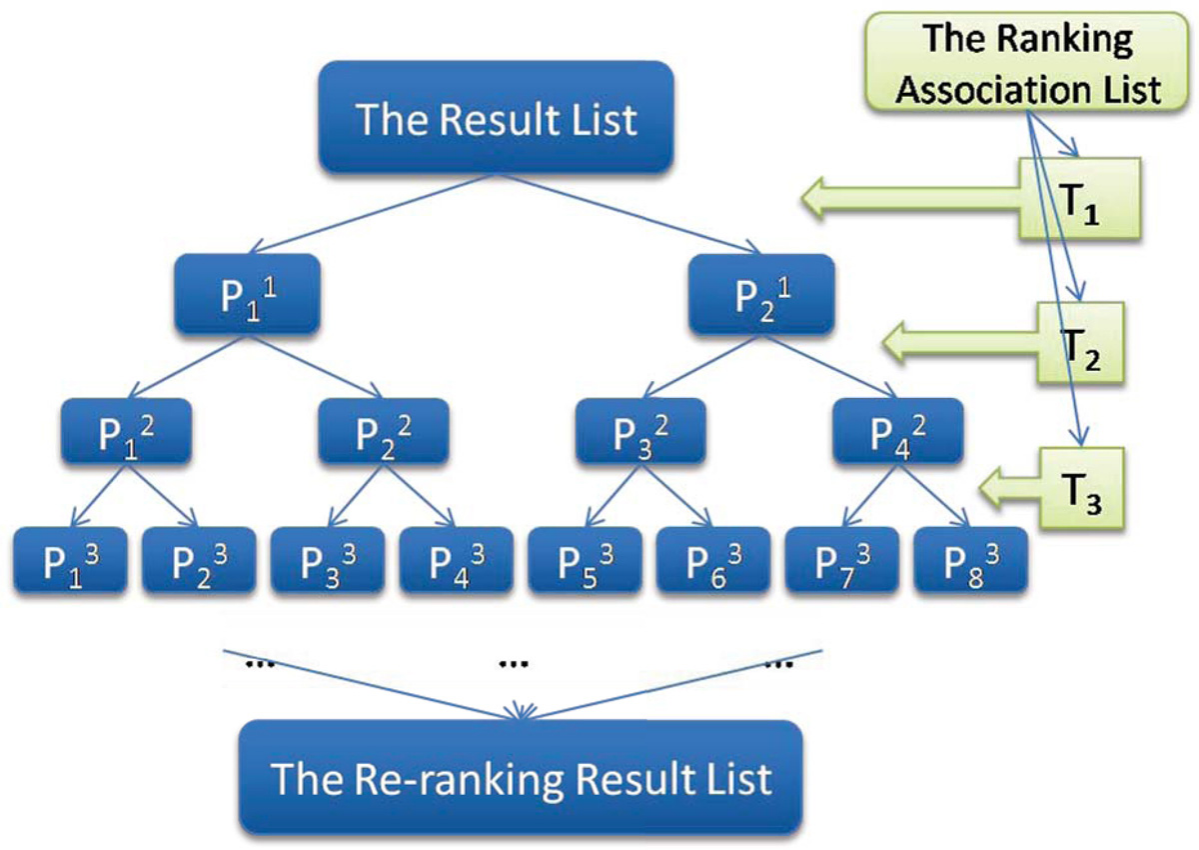
**Procedure of recursive re-ranking**. A recursive re-ranking algorithm is called for the phase of re-ranking in the section of the factor analysis based algorithm. The recursive division divides the passages into the base cases, according to the sorted term association, which is very similar to a binary tree. For example, the factor analysis based model gives a ranking list of terms as {*T*_1_, *T*_2_, *T*_3_} for re-ranking. The baseline results are then first re-ranking by *T*_1_, where $P_{1}^{1}$ are results containing *T*_1 _and $P_{2}^{1}$ are results not containing *T*_1_. Second, $P_{1}^{1}$ and $P_{2}^{1}$ are recursively re-ranked by *T*_2_. $P_{1}^{2}$ are the results containing *T*_2 _and *T*_1_, while $P_{2}^{2}$ are those not containing *T*_2 _but containing *T*_1_. $P_{3}^{2}$ are the results containing *T*_2 _but not containing *T*_1_, while $P_{4}^{2}$ are those not containing *T*_2 _and *T*_1_. Similarly, $P_{1}^{2}$(*i *= {1, 2, 3, 4} are re-ranked by *T*_3 _at the third step.

There are three main phases. The phase of **Initialization **gives the initial values i.e. *k *= 10, which we will give a deep discussion in the section of influence of K for recursive re-ranking. The phase of **Recursive division **divides the passages into the base cases, according to the ranking association. This procedure is displayed in Figure 9, which is very similar to a binary tree. For example, the factor analysis based model gives a ranking list of terms as {*T*_1_, *T*_2_, *T*_3_} for re-ranking. The baseline results are then first re-ranking by *T*_1_, where $P_{1}^{1}$ are results containing *T*_1 _and $P_{2}^{1}$ are results not containing *T*_1_. Second, $P_{1}^{1}$and $P_{2}^{1}$ are recursively re-ranked by *T*_2_. $P_{1}^{2}$are the results containing *T*_2 _and *T*_1_, while $P_{2}^{2}$ are those not containing *T*_2 _but containing *T*_1_. $P_{3}^{2}$are the results containing *T*_2 _but not containing *T*_1_, while $P_{4}^{2}$ are those not containing *T*_2 _and *T*_1_. Similarly, $P_{1}^{2}$(*i *= {1, 2, 3, 4} are re-ranked by *T*_3 _at the third step. Finally, the phase of **Re-ranking **gets the passages in the base cases $P_{i}^{k}\left(i=1,..,2^{k}\right).$Finally, a recursive result list for re-ranking is generated.

### Related work

Modelling and mining term association is important for information retrieval, which allows an IR system given a user's query terms to retrieve relevant documents more precisely.

Metzler and Croft [18] developed a general, formal framework for modelling term dependencies via Markov random fields. They not only made used of features based on occurrences of single terms, ordered phrases, and unordered phrases, but also explored full independence, sequential dependence and full dependence variant of the model. In addition, the training data were needed in the model for the parameters. Their ad hoc retrieval experiments showed improvements by modeling dependencies, especially on the larger collections.

Deerwester et al [19] proposed an approach to automatic indexing and retrieval, which was to take advantage of implicit higher-order structure in the association of terms with documents in order to improve the detection of relevant documents on the basis of terms found in queries. The proposed approach tried to overcome the deficiencies of term-matching retrieval by treating the unreliability of observed term-document association data as a statistical problem. They assumed that some underlying latent semantic structure in the data was obscured by the randomness of word choice with respect to retrieval. Then, they use statistical techniques to estimate the latent semantic structure for indexing and retrieval. 

Grefenstette [20] proposed an extraction technique using coarse syntactic analysis without domain knowledge, which produced word associations as lists of words related to the work appearing in a corpus. Their experimental results confirmed that, when the closest related terms were used in query expansion of a standard information retrieval data set, the results were much better than that given by document co-occurrence techniques, and slightly better than using unexpanded queries.

Hiroyuki Kaji at el [21] presented a method for automatically generating a corpus-dependent association thesaurus from a text corpus. This method consisted of extracting terms and co-occurrence data from a corpus and analysing the correlation between terms statistically. They conducted the experiments on a newspaper article corpus, which proved that the thesaurus navigator efficiently explored information through a text corpus when the information needs were vague.

Manna and Gedeon [22] proposed a term association model which extracted significant terms as well as the important regions from a single document, which based on the subjective data analysis without predefined knowledge. They claimed that the model overcame the basic drawback of existing language models for choosing significant terms in single documents.

Wei et al [23] proposed a technique using association rule mining for the discovery of the associations which took in account not only the co-occurrence frequency but also the confidence and direction of the association rules. They consistently improved the effectiveness of the retrieval over the set of 48 test queries on the Associated Press 1990 news wires corpus of the TREC4 benchmark by query expansion using term association rules.

In this work, we propose a term association approach to customize a factor analysis based model to quantify the importance and reliance of term associations. Independent keywords, disordered dependent phases and high-order structure are considered at the same time in the proposed approach. In addition, we focus on the appearance of the terms at the same context statistically but not the distance among the terms.

As a popular analysis method, factor analysis is attractive in IR for two main reasons. One apparent advantage of factor analysis is that users can use it to reduce the dimensionality of the data. The other one is to find the hidden patterns. Mandl [24] discussed methods for dimensionality reduction using factor analysis in IR. Machado at el [25] presented a perspective to image retrieval based on multivariate factor analysis to minimize data redundancy and reveal hidden patterns. Mehta at el [26] proposed an approach for cross-system personalization by factor analysis. Their proposed factor analysis method offered an algorithmic improvement over their previous work by taking into account the incompleteness of data. In our proposed approach, factor analysis is applied to discover some hidden common factors as the "eliteness" variables that can be used to estimate the importance of term associations.

Some related work has been done in the biomedical domain during the past few years. We investigated the optimization of multiple sources in [27], where a robust approach to optimizing multiple sources has been proposed. The proposed approach in the metasearch system has access to the baselines from three IR models as DFR, BM25 and language model. In [16], we concentrated on passage extraction and result combination. Three algorithms are presented for passage extraction to build indices and two result combination methods are proposed to combine the retrieval results from different indices. A naive model using factor analysis was also applied to improve the baselines for result combination, where unigrams and bigrams are considered. We also studied on a Bayesian learning approach to promoting diversity in ranking in [28]. In this approach, a re-ranking model computed the maximum posterior probability of the hidden property corresponding to each retrieved passage. Then it iteratively groups the passages into subsets according to their properties. In this paper, we focus on modelling term associations. The latent factors behind term associations reflect the importances and reliance of these term associations. They are decided by the proposed factor analysis based model and their term appearances in the first round retrieved passages.

Principal Component Analysis (PCA) [29] and factor analysis are two methods that can help reveal simpler patterns within a complex set of variables. In particular, they seek to discover if the observed variables can be explained largely or entirely in terms of factors. The main commonality between PCA and factor analysis is that they both have eigenvectors, eigenvalues, loading factors and scores. The differences are: (1) PCA is often used as a simple starting point in multivariate analysis; (2) factor analysis is often considered to be "statistical" in nature rather than purely mathematical as in PCA, since PCA eigenvectors cumulatively account for all the variability in the data set whereas factor analysis results include an unresolved component; (3) factor analysis results are often transformed through varimax and other methods to optimize eigenvectors for interpretation. This motivates us to choose factor analysis to compute the importance and reliance of term associations, in order to find the hidden "eliteness" variables. An n-gram [30] is a subsequence of *n *items from a given sequence. An n-gram model is a type of probabilistic model for predicting the next item in such a sequence. Some language models built from n-grams are "(*n - *1)-order Markov models". Its grammar is a representation of an *n^th ^*order Markov model in which the probability of occurrence of a symbol is conditioned upon the prior occurrence of (*n - *1) other symbols. Probabilistic latent semantic analysis (PLSA) [31] is a method of latent semantic analysis that uses probabilistic means to obtain the hidden topics and their relationships to terms and documents. In this paper, we use factor analysis to estimate the latent factors and compute the communalities for term associations statistically.

We adopt the Generalized Sequential Pattern (GSP) algorithm [32] as a comparison to our proposed approach, which contains two main steps as candidate generation and support counting. At first, all single items (1 - *sequences*) are counted. Then, from the frequent single items, a set of candidates of 2 - *sequences *are formed and filtered to identify their frequencies by removing the non-frequent items based on the minimum support. The frequent 2 *- sequences *are used to generate the candidates of 3 *- sequences*. This process is repeated until no more frequent sequences are found. The support counting is based on the minimum support value.

## Competing interests

The authors declare that they have no competing interests.

## Authors' contributions

This is a featuring work done by QH as a part of her Ph.D. thesis. JXH supervised the project and revised the manuscript. JXH and XH contributed in the study design and experiments. All authors read and approved the final manuscript.
